# Regulation of the *Candida albicans* Hypha-Inducing Transcription Factor Ume6 by the CDK1 Cyclins Cln3 and Hgc1

**DOI:** 10.1128/mSphere.00248-16

**Published:** 2017-03-08

**Authors:** Sigal Mendelsohn, Mariel Pinsky, Ziva Weissman, Daniel Kornitzer

**Affiliations:** Department of Molecular Microbiology, B. Rappaport Faculty of Medicine, Technion—I.I.T. and the Rappaport Institute for Research in the Medical Sciences, Haifa, Israel; University of Texas Health Science Center

**Keywords:** *Candida albicans*, Cdc4, Cln3, Hgc1, SCF, morphogenesis

## Abstract

The ability to switch between proliferation as yeast cells and development into hyphae is a hallmark of *Candida albicans*. The switch to hyphal morphogenesis depends on external inducing conditions, but its efficiency is augmented in stationary-phase cells. Ume6, a transcription factor that is itself transcriptionally induced under hypha-promoting conditions, is both necessary and sufficient for hyphal morphogenesis. We found that Ume6 is regulated posttranslationally by the cell cycle kinase Cdc28/Cdk1, which reduces Ume6 activity via different mechanisms using different cyclins. Together with the cyclin Hgc1, Cdk1 promotes degradation of Ume6 via the SCF^CDC4^ ubiquitin ligase. Since *HGC1* is a key transcriptional target of Ume6, this results in a negative-feedback loop between Hgc1 and Ume6. In addition, we found that Cln3, a G_1_ cyclin that is essential for cell cycle progression and yeast proliferation, suppresses hyphal morphogenesis and that Cln3 suppresses Ume6 activity both in the heterologous *Saccharomyces cerevisiae* system and in *C. albicans* itself. This activity of Cln3 may provide the basis for the antagonistic relationship between yeast proliferation and hyphal development in *C. albicans*.

**IMPORTANCE** The yeast to hypha (mold) morphogenetic switch of *Candida albicans* plays a role in its virulence and constitutes a diagnostic trait for this organism, the most prevalent systemic fungal pathogen in industrialized countries. It has long been known that hyphae are most efficiently induced from stationary cultures. Here, a molecular basis for this observation is provided. The G_1_ cyclin Cln3, an essential promoter of yeast proliferation, was found to suppress hyphal induction. Suppression of hyphal induction is achieved by inhibition of the activity of the central activator of hyphal morphogenesis, the transcription factor Ume6. Thus, levels of Cln3 control the switch between proliferation of *C. albicans* as individual yeast cells and development into extended hyphae, a switch that may preface the proliferation/differentiation switch in multicellular organisms.

## Abstract (precis)

The yeast to hypha (mold) morphogenetic switch of *Candida albicans* plays a role in its virulence and constitutes a diagnostic trait for this organism, the most prevalent systemic fungal pathogen in industrialized countries. It has long been known that hyphae are most efficiently induced from stationary cultures. Here, a molecular basis for this observation is provided. The G_1_ cyclin Cln3, an essential promoter of yeast proliferation, was found to suppress hyphal induction. Suppression of hyphal induction is achieved by inhibition of the activity of the central activator of hyphal morphogenesis, the transcription factor Ume6. Thus, levels of Cln3 control the switch between proliferation of *C. albicans* as individual yeast cells and development into extended hyphae, a switch that may preface the proliferation/differentiation switch in multicellular organisms.

## INTRODUCTION

*Candida albicans* is a human commensal fungus that can cause superficial infections in immunocompetent individuals, as well as life-threatening systemic infections in immunocompromised patients (1). *C. albicans* is able to assume different growth forms, most notably, yeast, hyphal, and pseudohyphal morphologies (2). This ability to switch between different modes of growth and proliferation appears to be important for virulence, based on the reduced pathogenicity in a mouse model of infection by mutants locked in the yeast mode (3, 4).

The cellular morphology is heavily dependent on growth conditions: whereas in standard rich medium at 30°C, wild-type *C. albicans* grows usually as yeast, many growth conditions have been identified that induce the switch to hyphal growth. These include an elevated temperature (37°C), elevated CO_2_, neutral pH, addition of serum, or various specific growth media that have little in common except that they often impose a growth limitation (reviewed in reference 5). Additional factors that can influence morphogenetic switching are the quorum-sensing molecules tyrosol, an inducer of hyphal growth (6), and farnesol, a repressor (7).

Genetic analysis has elucidated at least part of the regulatory pathways that link extracellular stimuli to morphogenesis. Several signal transduction pathways, notably, the mitogen-activated protein kinase (MAPK)-dependent (8) and cyclic AMP (cAMP)/protein kinase A (PKA)-dependent (9, 10) pathways, that participate in the induction of filamentation have been identified. A number of transcription factors were identified that can influence filamentous growth, including Cph1 (11), Efg1 (4, 12), Cph2 (13), *C. albicans* Tec1 (CaTec1) (14), CaRim101 (15), CaTup1 (16), CaNrg1 (17), CaMcm1 (18), CaFkh2 (19), and CaUme6 (20, 21). Some of these transcription factors were found to be targets of hypha-inducing signal transduction pathways (22, 23). Induction of filamentous morphology by extracellular signals is accompanied by a distinct transcription program with, notably, expression of genes encoding cell surface components such as the Hwp1, Ece1, and Als3 proteins (13, 24, 25).

Although several of the transcription factors mentioned above are important for hyphal growth, ectopic expression of any of these transcription factors by itself is unable to induce authentic hyphal growth. An exception is CaUme6, which was reported to induce hyphae upon overexpression (3). Ca*ume6*^*−*/*−*^ mutants transiently formed germ tubes when exposed to hypha-inducing conditions but were unable to sustain hyphal elongation and hypha-specific gene expression under all conditions tested (20, 21).

Cell morphogenesis is closely associated with regulation of the cell cycle, a link that is best understood in budding yeasts (26, 27). The morphogenetic switch in *C. albicans* may therefore be expected to involve regulation at the level of the cell cycle regulatory machinery as well (28, 29). The notion of a link between cell cycle and morphogenesis is supported by the observation that various treatments that inhibit cell cycle progression cause a switch to polarized growth (see, for example, references 30 and 31). Similarly, depletion of the Polo-like kinase CaCdc5, a mitotic regulator (30), and depletion of the *C. albicans* Cln3 homolog, an essential cyclin, were shown to induce polarized growth (32, 33). Notably, however, in all of the instances mentioned above, the polarized growth assumed pseudohyphal rather than typical hyphal morphologies (2), with the possible exception of Cln3 depletion. One mutation that induces true hyphal growth is the deletion of Ca*CDC4*, which encodes a substrate-recognition subunit of the SCF ubiquitin ligase (34, 35), the homolog of which is required for cell cycle progression in *Saccharomyces cerevisiae* (36, 37).

We had previously identified the cell cycle inhibitor Sol1 as a substrate of Cdc4. However, deletion of *SOL1* failed to suppress the hyphal phenotype of the Ca*cdc4*^*−*/*−*^ deletion, implying that one or more additional SCF^CaCDC4^ substrates are responsible for this phenotype. Here, we identified CaUme6 as the SCF^CaCDC4^ substrate that, together with Sol1, is responsible for the hyphal phenotype of Ca*cdc4*^*−*/*−*^. SCF^CDC4^ requires phosphorylation of its substrates for recognition (38). In a screen for the kinase responsible for CaUme6 degradation, we identified the CDK1 cyclin CaCln3 as a suppressor of CaUme6 activity. However, CaCln3 activity led to CaUme6 stabilization rather than degradation. In contrast, the CDK1 cyclin Hgc1 was required for CaUme6 degradation. Since *HGC1* is a key transcriptional target of CaUme6, this generates a negative-feedback loop in which CaUme6 activity causes its own demise. Suppression of CaUme6 activity by CaCln3 disrupts this feedback loop, leading to stabilization of CaUme6.

## RESULTS

### CaUme6 is a substrate of SCF^CaCDC4^.

**FIG 1:**
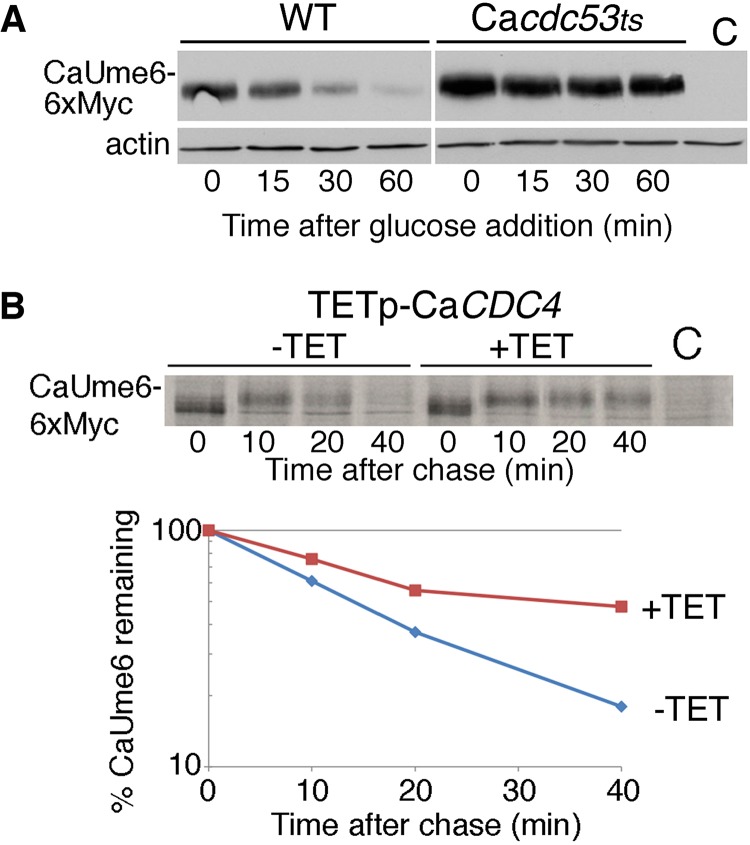
CaUme6 is stabilized in the absence of SCF^CaCDC4^ activity. (A) CaUme6-6xMyc was expressed from the *MAL2* promoter of plasmid KB2147 in either wild-type (WT; KC2) or Ca*cdc53*^−/*ts*^ (KC363) cells by inducing the cultures for 3 h with maltose. Aliquots were taken at the indicated times after glucose addition, and CaUme6 was visualized by Western blotting. C, no-tag control. (B) The TETp-Ca*CDC4* strain contains a single copy of the Ca*CDC4* gene under the regulation of the TEToff promoter; i.e., the promoter is shut off in the presence of tetracycline. Myc epitope-tagged CaUme6 was expressed from the *CUP1* promoter of plasmid KB1994 in strain KC200 (TETp-Ca*CDC4*). Degradation of CaUme6 was monitored by [^35^S]methionine pulse-chase analysis in a culture preincubated for 3 h with tetracycline versus a control culture. Both cultures were incubated for 15 min with 0.1 mM copper prior to labeling. For each time point, equal radioactivity counts were subjected to immunoprecipitation with anti-Myc antibodies, and the immunoprecipitate was loaded onto the gel. In the presence of tetracycline, a slower-migrating form of CaUme6 accumulates. The graph indicates the amount of CaUme6 signal at each time point, relative to the 0 time point. C, no-tag control.

The recognition sequence of Cdc4-type substrate receptors is not well defined but commonly includes a proline residue following the phosphorylated residue and a proline or other hydrophobic amino acid(s) preceding it (39). Analysis of the CaUme6 sequence identified many such potential recognition sites. To directly test whether CaUme6 degradation depends on the presence of SCF^CaCDC4^, we first tested the stability of CaUme6 in wild-type versus Ca*cdc53* mutant cells (40). CaCdc53 encodes the cullin subunit, an essential scaffold subunit common to all SCF complexes. As shown in Fig. 1A, CaUme6 was normally rapidly degraded upon promoter shutoff but was almost totally stabilized in the Ca*cdc53ts* mutant, suggesting that SCF activity is required for CaUme6 degradation. We next tested whether the F-box protein CaCdc4 was required for CaUme6 degradation by measuring CaUme6 stability by pulse-chase analysis in a strain depleted for CaCdc4. As shown in Fig. 1B, CaUme6 was rapidly degraded in the control strain, with a half-life of 15 min, whereas the CaCdc4-depleted strain showed stabilization of CaUme6, consistent with CaUme6 being a substrate of SCF^CaCDC4^.

### **Both CaUme6 and Sol1 contribute to the hyphal phenotype of Ca*cdc4***^*−*/***−***^**.**

**FIG 2:**
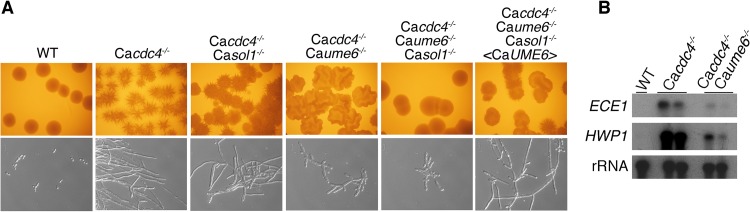
Genetic epistasis analysis of the *C. albicans cdc4* mutant together with the *sol1*^−/*−*^ and Ca*ume6*^*−*/*−*^ deletions. (A) Strains KC2 (wild type), KC138 (Ca*cdc4*^−/−^), KC196 (Ca*cdc4*^−/−^ Ca*sol1*^−/−^), KC449 (Ca*cdc4*^−/−^ Ca*ume6*^−/−^), KC462 (Ca*cdc4*^−/−^ Ca*sol1*^−/−^ Ca*ume6*^−/−^), and KC533 (Ca*cdc4*^−/−^ Ca*sol1*^−/−^ Ca*ume6*^−/−^ <Ca*UME6*>) were grown for 2 days at 30°C on yeast extract-peptone-dextrose (YPD) plates (top) or in liquid YPD to mid-log phase (bottom). The rightmost panels show the phenotype of the Ca*UME6* reintegrant, which was obtained by reintegration in the triple mutant of the Ca*UME6* open reading frame carried on a Ca*URA3*-marked plasmid at the Ca*UME6* locus. (B) The induction of hypha-specific genes by the deletion of Ca*CDC4* is largely restored by the further deletion of Ca*UME6*. The wild-type starting strain and the Ca*cdc4*^*−*/*−*^ and Ca*cdc4*^*−*/*−*^ Ca*ume6*^*−*/*−*^ mutants (two independently constructed strains each) were grown in regular YPD medium at 30°C. mRNA levels of the hypha-specific genes *ECE1* and *HWP1* were detected by Northern blotting. rRNA served as loading control.

In order to investigate the possibility that CaUme6 is the critical target of SCF^CaCDC4^, responsible for the hyphal phenotype of the Ca*cdc4*^*−*/*−*^ mutant (34), we performed genetic epistasis analysis using comparisons between the Ca*CDC4* and Ca*UME6* deletion mutants. If CaUme6 stabilization indeed caused the constitutively hyphal phenotype of Ca*cdc4*^*−*/*−*^, then deletion of Ca*UME6* in the Ca*cdc4*^*−*/*−*^ mutant should suppress this phenotype. As shown in Fig. 2, this was not the case: the double Ca*cdc4*^*−*/*−*^ Ca*ume6*^*−*/*−*^ mutant was still filamentous, albeit mostly pseudohyphal rather than hyphal. However, we knew from previous work that Sol1, another SCF^CaCDC4^ substrate, may also be involved in the filamentous growth of the Ca*cdc4*^*−*/*−*^ mutant (34). We therefore tested the deletion of both Ca*UME6* and *SOL1* together in the Ca*cdc4*^*−*/*−*^ background; the triple mutant lost its hyphal growth phenotype at the levels of both cell morphology and colony morphology. Reintroduction of a wild-type Ca*UME6* allele restored filamentous growth to the mutant (Fig. 2A), consistent with CaUme6 being (to a major extent if not exclusively) responsible for the hyphal phenotype of the Ca*cdc4*^*−*/*−*^ mutant. In addition to this morphological analysis, we also measured expression levels of two hypha-specific genes, *HWP1* and *ECE-1*, in the Ca*cdc4*^*−*/*−*^ strain versus the Ca*cdc4*^*−*/*−*^ Ca*ume6*^*−*/*−*^ strain. In the absence of Ca*UME6*, the increase in hypha-specific gene expression in the Ca*cdc4*^*−*/*−*^ strain was largely abolished, even in the presence of *SOL1* (Fig. 2B).

### Recapitulation of CaUme6 degradation in *S. cerevisiae*.

**FIG 3:**
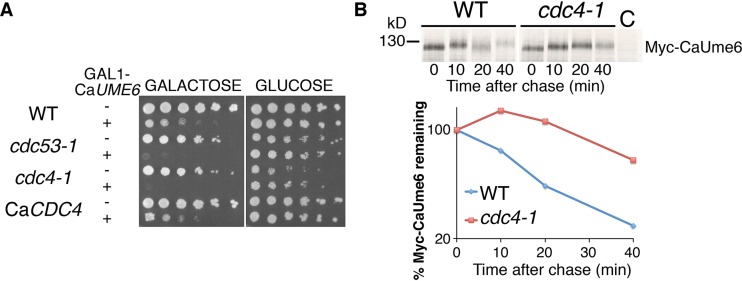
CaUme6 is recognized by SCF^CDC4^ in *S. cerevisiae*. (A) SCF^CDC4^ mutants are hypersensitive to CaUme6 overexpression. Fivefold dilutions of *S. cerevisiae* cells carrying the indicated mutation (KY337, KY440, KY442, or KY879) and harboring either a vector plasmid or a plasmid carrying Ca*UME6* under the regulation of the *GAL1* promoter (KB2028) were spotted on synthetic dropout plates with either glucose (*GAL1*-repressing) or galactose (*GAL1*-inducing) as the carbon source. Plates were incubated for 2 days (glucose) or 3 days (galactose) at 30°C. (B) CaUme6 is stabilized in the *S. cerevisiae cdc4-1* mutant. Degradation of epitope-tagged CaUme6 expressed from the *GAL1* promoter (plasmid KB2117) was monitored in the indicated strains by [^35^S]methionine pulse-chase analysis at 30°C. For each time point, equal radioactivity counts were subjected to immunoprecipitation with anti-Myc antibodies, and the immunoprecipitate was loaded onto the gel. The graph indicates the amount of CaUme6 signal at each time point, relative to the 0 time point. C, no-tag control.

F-box proteins of the Cdc4 type universally require phosphorylation of the substrate at one or multiple sites for recognition (36, 41–43). Consistent with the consequent assumption that CaUme6 requires phosphorylation for recognition by the SCF^CaCDC4^ ligase, a lower-mobility species of CaUme6 was seen to accumulate in CaCdc4-depleted cells (Fig. 1B). Thus, in order to understand the regulation of CaUme6 degradation, it was important to identify the kinase(s) that phosphorylates CaUme6 at the CaCdc4 recognition sites. Because of the difficulty of carrying out extensive genetic screens in *C. albicans*, we tested whether *S. cerevisiae* could serve as a model organism for the investigation of CaUme6 degradation. Strikingly, expression of Ca*UME6* under the control of the strong inducible *GAL1* promoter in *S. cerevisiae* was toxic, and this toxicity was exacerbated in the *cdc4-1* mutant and in *cdc53-1*, a mutant of the SCF cullin subunit (Fig. 3A). This hypertoxicity of CaUme6 correlated with partial stabilization of CaUme6 in the *cdc4-1* hypomorphic mutant (Fig. 3B). Interestingly, substituting *CDC4* with Ca*CDC4* in *S. cerevisiae* restored the strain to normal sensitivity to CaUme6 (Fig. 3A), suggesting that the CaUme6-CaCdc4 interaction could be reconstituted in *S. cerevisiae*.

### Identification of a potential CaUme6 kinase in *S. cerevisiae*.

**FIG 4:**
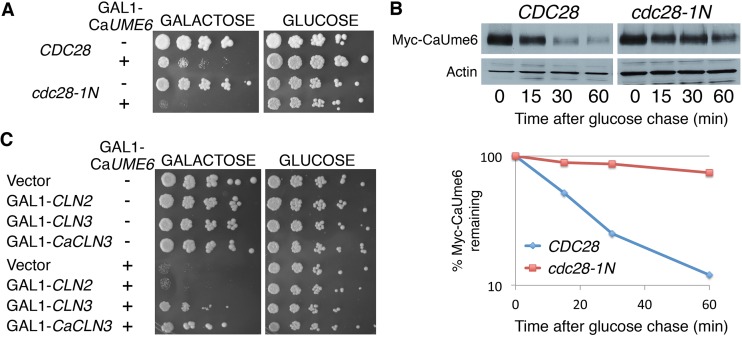
CaUme6 overexpression toxicity in *S. cerevisiae* CDK mutants and cyclin-overexpressing strains. (A) Ca*UME6* was expressed from plasmid KB2117 either in wild-type *S. cerevisiae* (KY337) or in a mutant of the main cell cycle CDK *CDC28* (KY414). The indicated strains were spotted and incubated as described for Fig. 3A, except the incubation was performed for 4 days at 24°C. (B) CaUme6 degradation was measured using the strains described in the panel A legend by shifting cells expressing CaUme6-6xMyc from galactose to glucose and following CaUme6 levels by Western blotting. The graph indicates for each time point the amount of CaUme6-6xMyc remaining relative to the 0 time point, normalized to the actin signal. (C) The G_1_ cyclins Cln2 (plasmid KB1826), Cln3 (KB991), and CaCln3 (KB2144) were co-overexpressed with CaUme6 (KB2117) as indicated and incubated were for 3 days (galactose) or 2 days (glucose) at 30°C.

The degradation of CaUme6 by SCF^CDC4^ or SCF^CaCDC4^ in *S. cerevisiae* implies that one or more kinases in that organism are capable of phosphorylating CaUme6. We therefore next addressed the identity of the CaUme6 kinase, using the toxicity phenotype of CaUme6 overexpression in *S. cerevisiae* as the initial assay. Since Cdc4 substrates are often phosphorylated by cyclin-dependent kinases (43), we started by assaying CaUme6 sensitivity in a mutant of the main cell cycle CDK, Cdk1/Cdc28. We found that *cdc28-1N* cells were hypersensitive to CaUme6 overexpression (Fig. 4A). Furthermore, CaUme6 was strongly stabilized in the *cdc28-1N* mutant (Fig. 4B).

Cdc28 in *S. cerevisiae* is activated by nine cyclins—three G_1_ cyclins and six B-type cyclins—which also contribute to the substrate specificity of the kinase (44). In other instances of CDK-mediated protein degradation, the overexpression toxicity of SCF substrates could be partially suppressed by co-overexpression of the cyclin required for their degradation (45, 46). We therefore next tested whether CaUme6 toxicity could be suppressed by overexpression of a Cdc28 cyclin. Whereas B-type cyclin overexpression did not suppress CaUme6 toxicity (data not shown), the G_1_ cyclin Cln3 (but not Cln2) was able to partly suppress CaUme6 toxicity when overexpressed (Fig. 4C, top panel). The *C. albicans* homolog of this gene, Ca*CLN3* (32, 33), was similarly able to partly suppress CaUme6 toxicity in *S. cerevisiae* (Fig. 4C, bottom panel).

### CaCln3 suppresses CaUme6-induced filamentation in *C. albicans*.

**FIG 5:**
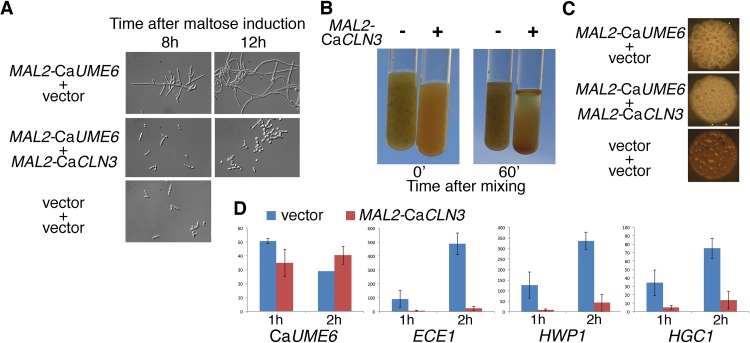
CaCln3 suppresses CaUme6 activity. (A) CaUme6 was ectopically expressed in *C. albicans* under the control of the maltose-inducible *MAL2* promoter (strain KC651), either alone or in the presence of overexpression of Ca*CLN3* (plasmid KB1831). The control strain was KC271. Stationary cell cultures were diluted into yeast extract-peptone (YEP)–2% maltose medium and photographed at the indicated times with a 40× objective and Nomarski optics. (B) Cultures of *C. albicans* expressing either CaUme6 alone (left tube) or CaUme6 in the presence of Ca*CLN3* overexpression (right tube) were grown overnight in YEP–2% maltose. The tubes were subjected to vortex mixing and photographed immediately or after standing for 60 min. (C) Suspensions of *C. albicans* cells expressing the indicated genes were inoculated onto a YEP–2% maltose agar plate and incubated 2 days at 30°C. The control strain was KC271. (D) Northern blotting of hypha-specific gene expression in strains overexpressing Ca*UME6* in the presence or absence of Ca*CLN3* overexpression. Cells were grown overnight in YEP–2% raffinose and then diluted in YEP–2% maltose. Aliquots for RNA extraction were taken 1 and 2 h after maltose induction. The transcript intensities (arbitrary values) were obtained by measuring the band intensities by the use of a phosphorimager. The gene-specific signals were normalized to the 18S rRNA signal for each lane. A total of 2 to 4 clones were tested for each condition; the bar graph indicates the average value, with the error bars indicating the variance. The original Northern blots are displayed in Fig. S1.

Ectopic overexpression of Ca*UME6* can induce hyphal growth, even in rich medium (20, 21). To test whether the suppression of CaUme6 by CaCln3 can be recapitulated in *C. albicans*, we assayed the effect of ectopic overexpression of CaCln3 on the CaUme6-induced hyphal growth. While Ca*UME6* expression under the control of the *MAL2* promoter indeed induced robust hyphal growth, co-overexpression of Ca*CLN3* suppressed this effect in a large measure and left the cells for the most part in the yeast morphology (Fig. 5A). This microscopic morphology was mirrored in the sedimentation of the culture: while elongated cells typically sediment faster than yeast cells, the cells in a culture overexpressing Ca*UME6* formed extended hyphae to such an extent that a never-sedimenting mycelium was formed in the test tube (Fig. 5B). In contrast, cells co-overexpressing Ca*CLN3* with Ca*UME6* sedimented normally. A corresponding effect was also seen at the level of colony morphology: the crenellated morphology, characteristic of filamentous growth, induced by CaUme6 was largely suppressed by co-overexpression of CaCln3 (Fig. 5C). Finally, we concomitantly tested expression of the hypha-induced genes *HWP1*, *ECE1*, and *HGC1* by Northern blotting in cells overexpressing Ca*UME6*, alone or together with Ca*CLN3*. All three genes were strongly induced upon Ca*UME6* induction alone, but this induction was strongly suppressed by co-overexpression of Ca*CLN3* (Fig. 5D). Ca*UME6* expression was barely affected by Ca*CLN3* co-overexpression, confirming that the effect of CaCln3 on CaUme6 is posttranscriptional.

10.1128/mSphere.00248-16.1 FIG S1  The Northern blots from which the quantitation displayed as bar graphs in Fig. 5D was derived. Band intensities were measured by the use of a phosphorimager. Each band was normalized to the 18S rRNA intensity in the same lane. The lane marked with a red asterisk and the band marked with a black asterisk were not included in the quantitation. V, vector. Download FIG S1, JPG file, 1.9 MB. Copyright © 2017 Mendelsohn et al. 2017 Mendelsohn et al. This content is distributed under the terms of the Creative Commons Attribution 4.0 International license.

### Role of Cdc28 cyclins in CaUme6 degradation.

**FIG 6:**
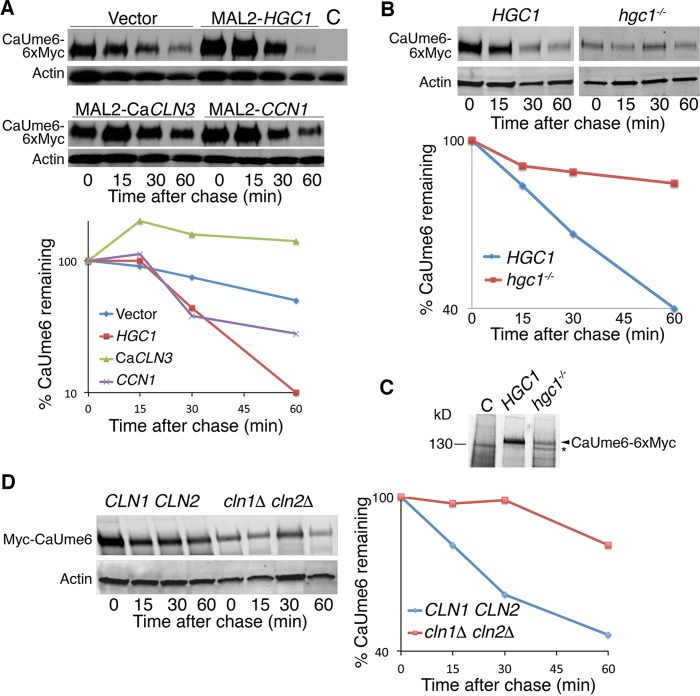
Hgc1 is responsible for CaUme6 degradation. (A) CaUme6-6xMyc was ectopically expressed in *C. albicans* under the control of the doxycycline-inducible Tet-on promoter of plasmid KB2270, either alone or together with overexpression under the control of the *MAL2* promoter of *HGC1*, Ca*CLN3*, or *CCN1* (plasmids KB1615, KB1697, and KB1698). Cells were shifted from YEP-raffinose to YEP–maltose–50 µg/ml doxycycline for 3 h and then washed three times with the same medium without doxycycline, and CaUme6-6xMyc levels were followed by Western blotting. (B) CaUme6 was ectopically expressed in *C. albicans* under the control of the *MAL2* promoter of plasmid KB2147 either in the wild-type strain (KC274) or in the *hgc1*^−/*−*^ strain (KC532). Cells grown in YEP-raffinose were induced for 2 h with 2% maltose, and then 2% glucose was added and CaUme6-6xMyc levels were followed by Western blotting. (C) Pulse-labeling of CaUme6 ectopically expressed in *C. albicans* under the control of the *MAL2* promoter of plasmid KB2147 either in the wild-type strain (KC965) or in the *hgc1*^−/*−*^ strain (KC1014). Cells were shifted from raffinose to maltose for 2 h and then washed in labeling medium and subjected to pulse-labeling with [^35^S]methionine for 8 min. CaUme6-6xMyc was immunoprecipitated with anti-Myc. The asterisk indicates a nonspecific band. (D) CaUme6 was ectopically expressed in *S. cerevisiae* under the control of the galactose-inducible *GAL1* promoter of plasmid KB2117, either in the wild-type strain (W303) or in the *cln1Δ cln2Δ* strain (KY387). The graphs indicate for each time point the amount of CaUme6-6xMyc remaining relative to the 0 time point, normalized to the actin signal.

Following the suppression of CaUme6 activity by CaCln3, we tested whether CaCln3 overexpression induces CaUme6 degradation. Unexpectedly in view of the previous results, rather than causing CaUme6 degradation, co-overexpression of CaCln3 caused stabilization of CaUme6 (Fig. 6A). This occurred in spite of the fact that in the same cells, co-overexpression of Ca*CLN3*—but not of other G_1_ cyclins—with Ca*UME6* caused suppression of the hyphal induction (see Fig. S2 in the supplemental material). Since Cdc28/Cdk1 had been found to be involved in CaUme6 degradation in *S. cerevisiae*, we screened additional *Candida albicans* Cdc28 cyclins for effects on CaUme6 degradation. Among the cyclins tested, only Hgc1 overexpression induced an acceleration of CaUme6 degradation (Fig. 6A). Conversely, a mutant lacking *HGC1* showed almost complete stabilization of CaUme6, placing the Cdc28 cyclin Hgc1 within the degradation pathway of CaUme6 (Fig. 6B) (a mutant lacking Ca*CLN3* was not tested since that mutant was inviable). Surprisingly, alongside stabilization of CaUme6, the *hgc1*^−/*−*^ mutant also exhibited markedly reduced steady-state levels of CaUme6. We tested whether this was due to reduced mRNA expression in the *hgc1*^−/*−*^ strain. However, levels of CaUme6 mRNA expressed under the control of the *MAL2* promoter were very similar in the wild-type and *hgc1*^−/*−*^ backgrounds (Fig. S3). We next tested whether this was an idiosyncrasy of our specific *hgc1*^−/*−*^ mutant strain by constructing a new *hgc1*^−/*−*^ mutant by clustered regularly interspaced short palindromic repeat (CRISPR) mutagenesis (47). In this new strain set, CaUme6 was still stabilized but showed lower expression levels. In order to test whether these lower levels were due to lower translation levels, cells were subjected to pulse-labeling with [^35^S]methionine (Fig. 6C). In three independent experiments, levels of CaUme6 were 65% ± 15% lower in the *hgc1*^−/*−*^ mutant than in the wild-type strain, suggesting that, in addition to promoting CaUme6 degradation, Hgc1 promotes CaUme6 translation.

10.1128/mSphere.00248-16.2 FIG S2  Only CaCln3 suppresses the hyphal induction by CaUme6. CaUme6 was ectopically expressed in *C. albicans* under the control of the doxycycline-inducible Tet-on promoter of plasmid KB2270, either alone or together with overexpression under the control of the *MAL2* promoter of *HGC1*, Ca*CLN3*, or *CCN1* (plasmids KB1615, KB1697, and KB1698). Cells were shifted from YEP-raffinose to YEP–maltose–50 µg/ml doxycycline for 24 h at 30°C and then visualized with a 40× objective and Nomarski optics. Download FIG S2, JPG file, 1.3 MB. Copyright © 2017 Mendelsohn et al. 2017 Mendelsohn et al. This content is distributed under the terms of the Creative Commons Attribution 4.0 International license.

10.1128/mSphere.00248-16.3 FIG S3  Effect of deletion of *HGC1* on CaUme6 mRNA levels expressed under the control of the *MAL2* promoter of KB2147. *HGC1* wild-type (KC274) and mutant (KC554) cells were grown in 2% raffinose and then induced with 2% maltose, and aliquots were taken at the indicated times after maltose induction. CaUme6-6xMyc levels were detected by Northern blotting and normalized to 18S rRNA. C, control cells harboring a vector plasmid only. Download FIG S3, JPG file, 0.4 MB. Copyright © 2017 Mendelsohn et al. 2017 Mendelsohn et al. This content is distributed under the terms of the Creative Commons Attribution 4.0 International license.

The stabilization of CaUme6 observed in *C. albicans hgc1*^−/*−*^ prompted us to revisit degradation of CaUme6 in *S. cerevisiae*. By sequence alignment, the closest homologs in *S. cerevisiae* to *C. albicans* Hgc1 are the G_1_ cyclins Cln1 and Cln2 (48, 49). We had found that Cln2 overexpression was unable to suppress CaUme6 toxicity in *S. cerevisiae* (Fig. 4C). Indeed, in *S. cerevisiae*, neither overexpression of Hgc1 nor overexpression of Cln2 induced any acceleration of CaUme6 degradation (Fig. S4). However, in the reciprocal experiment, deleting both the *CLN1* and *CLN2 HGC1* homologs from the *S. cerevisiae* genome resulted in almost complete stabilization of CaUme6 (Fig. 6D). This suggests that the kinase responsible for SCF^CDC4^-mediated CaUme6 degradation in *S. cerevisiae* is Cdc28-Cln1/2, the closest homolog of *C. albicans* Cdc28-Hgc1.

10.1128/mSphere.00248-16.4 FIG S4  Overexpression of Cln2 and Hgc1 does not accelerate CaUme6 degradation in *S. cerevisiae*. CaUme6 was ectopically expressed in *S. cerevisiae* under the control of the galactose-inducible *GAL1* promoter of plasmid KB2117 in the presence of a vector plasmid or the GAL1-*CLN2* KB1826 plasmid or the GAL1-*HGC1* KB2139 plasmid. Cells were shifted from synthetic raffinose medium to galactose for 3 h. Glucose was then added to reach a 2% concentration, and the levels of CaUme6 were followed by Western blotting. The graph indicates for each time point the amount of CaUme6-6xMyc remaining relative to the 0 time point, normalized to the actin signal. Download FIG S4, JPG file, 0.6 MB. Copyright © 2017 Mendelsohn et al. 2017 Mendelsohn et al. This content is distributed under the terms of the Creative Commons Attribution 4.0 International license.

## DISCUSSION

The observation that a mutant of Ca*CDC4*, one of the substrate recognition factors of the SCF ubiquitin ligase, is locked in the hyphal morphology demonstrated the involvement of ubiquitin-mediated protein degradation in *C. albicans* morphogenesis (34). Here, we identify a key hyphal morphogenesis transcription factor, CaUme6, as the critical SCF^CaCDC4^ substrate responsible for the hyphal phenotype of the Ca*cdc4*^*−*/*−*^ mutant. Like all known SCF^CDC4^ substrates, CaUme6 was expected to require phosphorylation in order to be recognized by its ubiquitin ligase. We identify here the Cdc28 cyclin Hgc1 and, in *S. cerevisiae*, the Cdc28 kinase, together with the G_1_ cyclins Cln1 and Cln2, as kinases that are required for CaUme6 degradation.

**FIG 7:**
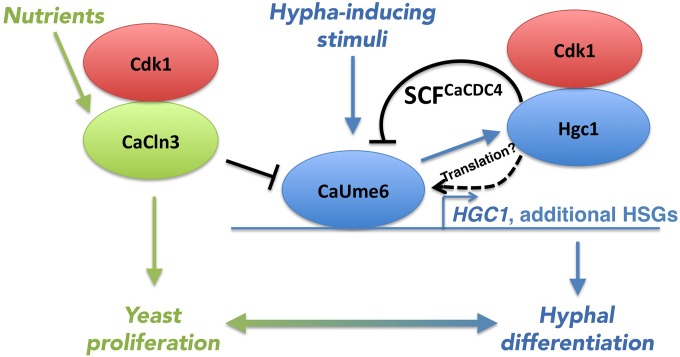
Model of the regulation of CaUme6 by Cdk1 (Cdc28) with the Hgc1 and CaCln3 cyclins. (Right side, blue color) CaUme6 induces *HGC1* alongside additional hypha-specific genes (HSGs), and Hgc1 together with Cdk1 induces SCF^CaCDC4^-mediated degradation of CaUme6. Hgc1 also stimulates CaUme6 translation via an unknown pathway. (Left side, green color) The inducer of proliferation CaCln3 is activated by nutrients and represses the activity of CaUme6 and thus hypha formation. Black, new regulations of CaUme6 identified in the present study.

The *C. albicans* G_1_ cyclin Hgc1 was shown to be absolutely necessary for hyphal growth (49). Specific substrates phosphorylated by Hgc1 under hyphal growth conditions include the septin Cdc11 (50), the Cdc42 GTPase-activating protein (GAP) Rga2 (51), and the transcription factor Efg1 (52). *HGC1* is an essential transcriptional target of CaUme6 in the hyphal induction pathway (53). Thus, the Hgc1-mediated degradation of CaUme6 leads to a negative-feedback loop that keeps cellular CaUme6 levels in check (Fig. 7), similarly to the negative-feedback loop between the transcription factor Gcn4 and the cyclin Pcl5 in both *C. albicans* and *S. cerevisiae* (45, 46). Complicating the picture, however, we also found a stimulatory effect of Hgc1 on CaUme6 translation, via an unknown mechanism. The translational effect shown here does not depend on the extended 5′ untranscribed region (5′ UTR) of the native Ca*UME6* transcript (54), since this region is absent from our *MAL2* promoter-driven Ca*UME6* expression construct. Thus, Hgc1 exerts both positive and negative effects on CaUme6 levels. Further elucidation of the mechanism of CaUme6 translational regulation by Hgc1 will be required to understand how Hgc1 affects net CaUme6 levels under different conditions.

CaUme6 degradation was previously found to be regulated by external conditions such as high CO_2_ and low O_2_ concentrations, in part via binding of the C terminus of Ofd1 (55). However, the negative-feedback regulation described here is operative in rich media under nonfilamentous growth conditions and appears thus to be distinct from this previously described regulation of CaUme6 degradation by CO_2_ and O_2_.

In the course of our search for the Cdc28 cyclin involved in CaUme6 degradation, we identified Cln3 as a cyclin that antagonizes CaUme6 activity. In the heterologous *S. cerevisiae* system, the toxicity of CaUme6 was suppressed by Cln3/CaCln3 overexpression. In *C. albicans*, in the presence of elevated CaCln3 levels, the ectopic induction of hyphal growth by CaUme6 overexpression was abolished. It is worth mentioning that under standard physiological hyphal induction conditions (37°C, 10% serum), overexpression of Ca*CLN3* also suppressed hyphal morphogenesis (T. Gildor and D. Kornitzer, unpublished data). The suppression of the CaUme6 phenotypes by CaCln3 in *C. albicans* was accompanied by a dramatic reduction in the CaUme6-induced expression of its target genes. The reduction in CaUme6 activity was also accompanied by a strong stabilization of CaUme6, which was initially surprising. However, the identification of the CaUme6-Hgc1 negative-feedback loop immediately suggests a solution to this apparent paradox: if CaCln3 affects CaUme6 transcriptional activity, then it would also disrupt the CaUme6-Hgc1 feedback loop by interfering with expression of *HGC1*, causing stabilization of CaUme6 (Fig. 7).

If CaCln3 suppresses CaUme6 transcriptional activity, then deletion of Ca*CLN3* should lead to increased CaUme6 activity. Ca*CLN3* is an essential gene and cannot be deleted; however, when Ca*CLN3* was placed under the control of a repressible promoter, it was found that yeast cells depleted of CaCln3 arrested in G_1_, grew in size, and eventually formed hypha- or pseudohypha-like extensions (32, 33). Interestingly, CaCln3-depleted cells were found to exhibit increased *HGC1* expression, which is consistent with an increase in CaUme6 activity (33).

The simplest explanation for the effect of *HGC1* and Ca*CLN3* on CaUme6 activity and stability is that Cdc28-Hgc1 and Cdc28-CaCln3 directly phosphorylate CaUme6. We were, however, unable to produce sufficient recombinant full-length CaUme6 to reconstitute these reactions *in vitro*. Instead, we were able to reconstitute in *S. cerevisiae* both the Cdc28-Cln1/2 dependence of CaUme6 degradation and the suppression of CaUme6 activity by Cdc28-Cln3/CaCln3. Reconstitution of these effects in a heterologous organism, in the absence of additional *C. albicans* factors, reinforces the assumption of direct phosphorylation of CaUme6 by these kinases in *S. cerevisiae* and, consequently, by their homologs Cdc28-Hgc1 and Cdc28-CaCln3 in *C. albicans*. Nonetheless, the possibility of an indirect effect of these kinases on CaUme6 via a third factor present in both organisms cannot be excluded.

The transcription factor CaUme6 lies at the heart of hyphal regulation in *C. albicans* as a necessary and sufficient factor for hyphal growth (3, 20) and as a key transcriptional target for the various signal transduction pathways that mediate hyphal induction stimuli (21). Here, we show that in addition to its transcriptional regulation, CaUme6 is also regulated posttranscriptionally by CaCln3. Whereas the transcriptional regulation of Ca*UME6* mediates the response to extracellular signals that induce hyphal growth, the CaCln3-mediated regulation may represent the input of cellular physiology in the dimorphic switch decision. The notion that cellular physiology affects responsiveness to hyphal induction is not new: for example, it was shown that stationary cells released in fresh hyphal induction medium exhibit more robust hyphal morphogenesis than mid-log-phase cells (24). This phenomenon could be explained in part by release from the inhibition mediated by the quorum-sensing molecule farnesol but likely involves cell-autonomous effects as well. However, the mechanism for this link between cell physiology and hyphal induction was unknown.

Our observation that CaCln3 suppresses CaUme6 activity provides a plausible mechanistic explanation for the antagonistic relationship between yeast proliferation and hyphal morphogenesis. Cell proliferation in all organisms is regulated by nutrient availability. The *S. cerevisiae* ortholog of CaCln3, Cln3, is the most upstream regulator at the start of the cell cycle (56, 57) and is subject to several transcriptional and posttranscriptional regulations linking its levels to the nutritional state of the cell (58–60). The regulation of Ca*CLN3* has not been investigated, but as an essential regulator of cell proliferation, it is likely to be similarly regulated by the physiological state of the cell. Thus, by responding inversely to CaCln3 levels, CaUme6 activity would be lower under optimal growth conditions and higher under nutrient-limiting conditions.

The proliferation-differentiation antagonism is well established in animal cells (61). In particular, cyclin D1, the functional homolog of fungal Cln3, has been widely shown not only to promote proliferation but also to inhibit epithelial differentiation (62) as well as myogenesis and neurogenesis (63). To the extent that the switch between proliferation as yeast cells and differentiation into hyphal cells prefaces differentiation pathways in higher organisms, the role of Cln3 in hyphal morphogenesis may mirror the antagonistic role of the cell cycle apparatus, and of cyclin D in particular, in multicellular differentiation systems.

## MATERIALS AND METHODS

### Plasmids and strains. (i) Plasmids.

The Ca*UME6* deletion plasmids KB2022 and KB2023 were generated by cloning the 5′ region (position −640 to position +1; SacI-SpeI) and 3′ region (position +2537 to position +3315; HindIII-KpnI) into KB985 and KB986 (34), respectively. KB2028 is Ca*UME6* (position +1 to position +3315; HindIII-KpnI) cloned into p416-GalI (64). KB2073 contains the CaUME6 region from position −1500 to position +2900; NotI-KpnI) cloned into BES116 (65). KB2117 was constructed by first introducing the single Myc epitope sequence of KB1321 (34) into p415GAL1 (64) to generate KB1319; the Ca*UME6* sequence was then fused downstream of the epitope tag sequence of KB1319. KB1994 is Ca*UME6* cloned at BamHI-HindIII and fused to the Myc epitope of KB1321. KB2147 was constructed by first cloning the Ca*UME6* open reading frame (ORF) (position +1 to position +2529) at EcoRV-HindIII under the control of the *MAL2* promoter of BES119 (65), followed by introduction of the 6xMyc-Sc*CYC1* terminator sequence of KB1578 (34) at HindIII-KpnI downstream of Ca*UME6*. For KB2270, first, a Tet-on vector plasmid (KB1868) was constructed by introducing an XmaI-EcoRI-NotI multicloning site between SalI and BglII of pNIM6 (66), followed by cloning of Ca*UME6-*6xMyc-*CYC1t* amplified from KB2147 between SalI and NotI. KB1615, KB1697, and KB1698 were previously described (48). KB1831 is Ca*CLN3* cloned at ClaI-ApaI under the control of the *MAL2* promoter of KB1817 (67). KB991 is a *URA3* 2μ *GAL1-CLN3* plasmid obtained from Gerry Fink. KB1826 contains the *CLN2* open reading frame cloned at EcoRI-XbaI between the galactose (GAL) promoter of plasmid p415GalL (64) and the 3xMyc tag of plasmid KB891 (41). KB2144 contains the Ca*CLN3* sequence (position +1 to position +2083), PstI-XhoI, first cloned into p424-Gal1 (64) and then transferred as a Gal1-Ca*CLN3* fragment, at SacI-XhoI, to p416GalL (CEN *URA3*). KB2139 contains the *HGC1* open reading frame cloned at XbaI-EcoRI under the control of the *GAL1* promoter of plasmid p416-GalI (64).

### (ii) Strains.

The *C. albicans* strains are listed in Table 1. *C. albicans* deletion of Ca*UME6* was achieved by sequential deletion of both alleles using plasmids KB2022 and KB2023 to generate KC445. Ca*CDC4* was deleted in KC445 using plasmid KB1344 (34) to generate KC449. To generate KC462, Ca*UME6* was deleted in KC446 (ura- derivative of KC196 obtained by 5-fluoroorotic acid [5-FOA] selection). KC533 is a Ca*UME6* reintegrant strain obtained by transformation of KC464 (the ura3- derivative of KC462 obtained by 5-FOA selection) using plasmid KB2073 digested with SmaI. SmaI targets the plasmid to the promoter region of the deleted Ca*UME6* allele and reconstitutes the full gene. We found that targeting the KB2073 plasmid to the *ADE2* locus instead does not complement Ca*UME6*, suggesting that the sequences extending 1,500 nucleotides (nt) upstream of the translation start site are not sufficient to support full expression. This is consistent with analyses indicating that Ca*UME6* possesses exceptionally long 5′ UTR and promoter sequences (54, 68). KC651 contains the *MAL2* promoter of plasmid pFA-URA3-MAL2p (69) integrated upstream of the Ca*UME6* open reading frame by PCR-targeted recombination. KC965 was constructed by transforming KC2 with the nourseothricin-resistant (Nat^r^) CRISPR-associated gene 9 (CAS9) pV1025 plasmid (47) followed by removal of the Nat^r^ marker as described in reference 47. To generate KC1014, HGC1 was mutated by CRISPR (47) using a guide RNA corresponding to positions 125 to 144 on the antisense strand and a mutagenic oligonucleotide introducing an XhoI site and a frameshift at position 124. The *S. cerevisiae* strains (Table 2) were all in the W303 background. KY879 was generated by shuffling the Ca*CDC4* gene on plasmid KB1261 (34) into MTY1260 (W303 *ura3-1 his3-11 15 trp1-1cdc4*::*HIS3* <CEN *URA3 CDC4>*; M. Tyers).

**TABLE 1:** List of *C. albicans* strains

Name	Genotype	Reference or source
KC2 = CAI4	*ura3Δ*::*imm434*/*ura3Δ*::*imm434*	73
KC138	*ura3Δ*/*ura3Δ cdc4Δ*::*hisG*-*URA3-hisG*/*cdc4Δ*::*hisG*	34
KC196	*ura3Δ*/*ura3Δ sol1Δ*/*sol1Δ cdc4Δ*::*hisG*-*URA3-hisG*/*cdc4Δ*::*hisG*	34
KC200	*ura3Δ*/*ura3Δ ENO1*/*eno1*::*ENO1*-*tetR*-ScHAP4AD-3*HA-*ADE2 cdc4Δ*::*hisG*/Tr-*CDC4*-*SAT1*	34
KC271 = SN78	*ura3Δ*/*ura3Δ leu2Δ*/*leu2Δ*	74
KC274 = SN148	*ura3Δ*/*ura3Δ his1Δ*/*his1Δ leu2Δ*/*leu2Δ arg4Δ*/*arg4Δ*	74
KC363	KC271 *cdc53Δ*::*LEU2/cdc53-1*	40
KC445	*ura3Δ*/*ura3Δ ume6Δ*::*hisG*/*ume6Δ*::*hisG*	This work
KC449	KC445 *cdc4Δ*::*hisG*-*URA3-hisG*/*cdc4Δ*::*hisG*	This work
KC462	*ura3Δ*/*ura3Δ sol1Δ*/*sol1Δ cdc4Δ*/*cdc4Δ ume6Δ*::*hisG*-*URA3-hisG*/*ume6Δ*::*hisG*	This work
KC532	*ura3Δ*/*ura3Δ his1Δ*/*his1Δ leu2Δ*/*leu2Δ arg4Δ*/*arg4Δ hgc1Δ*::*HIS1*/*hgc1Δ*::*LEU2*	Yue Wang
KC533	*ura3Δ*/*ura3Δ sol1Δ*/*sol1Δ cdc4Δ*/*cdc4Δ ume6*Δ/*ume6*Δ *ADE2*/*ade2*::<*URA3 UME6*>	This work
KC651	KC271 *URA3 MAL2p*::*UME6*/*UME6*	This work
KC965	KC2 *ENO1*/*eno1*::*CaCAS9*	This work
KC1014	KC965 *hgc1−*/*−*	This work

**TABLE 2:** List of *S. cerevisiae* strains

Name	Genotype	Reference or source
KY337 = W303-1A	*MATa ura3-1 can1-100 GAL*^+^ *leu2-3*,*112 trp1-1 ade2-1 his3-11*,*15*	R. Rothstein
KY440 = MTY668	W303 *cdc4-1*	M. Tyers
KY442 = MTY740	W303 *cdc53-1*	M. Tyers
KY387	W303 *cln1*::*TRP1 cln2*::*LEU2*	75
KY414	W303 *ura3-1 leu2-3*,*112 trp1-1 cdc28-1N*	A. Amon
KY879	W303 *ura3-1 his3-11*, *15 trp1-1cdc4*::*HIS3* <CEN *TRP1* Ca*CDC4*>	This work

### mRNA analysis.

For RNA analysis by Northern blotting, a 10-ml volume of yeast culture was collected for each sample, harvested, and frozen in liquid nitrogen. The RNA was extracted according to the “hot phenol” method (70). A total of 3 μg of RNA was loaded in each lane. Radioactive DNA probes were synthesized using a NEBlot kit (New England Biolabs). The radioactive signals were quantitated with a phosphorimager. All specific gene signals were normalized to the 18S rRNA signal of the same gel lane.

### Protein analysis.

Protein levels were assayed by Western blotting using monoclonal antibody 9E10 to detect the Myc epitope. Proteins were extracted by the quantitative NaOH/2-mercaptoethanol method, as described previously (71). To compare steady-state protein levels, equal protein amounts were loaded; to monitor protein disappearance after promoter shutoff, equal culture volume equivalents were loaded. Loading and transfer were monitored by Ponceau staining of the membrane and by actin quantitation using an anti-β-actin antibody (AB8224; Abcam, Inc.). Quantitation was achieved either using horseradish peroxidase (HRP)-conjugated secondary antibodies, followed by detection of enhanced chemiluminescence (ECL) signals with a Bio-Rad Chemidoc apparatus, or using Li-COR infrared fluorescence IRDye secondary antibodies, followed by detection performed with an Odyssey imaging system. Pulse-chase analysis was performed essentially as described previously (72), except maltose was used for CaUme6-6xMyc induction.

### Microscopy.

Cells were fixed in 70% ethanol and visualized with a Zeiss AxioImager M1 microscope equipped with differential inference contrast (DIC) optics, using a 40× or 100× objective. Colonies were visualized with a Zeiss Stemi 2000C binocular microscope.
