# Supplementary figures and images for: Regulation of the Candida albicans Hypha-Inducing Transcription Factor Ume6 by the CDK1 Cyclins Cln3 and Hgc1
Source: mSphere. 2017 Mar 8;2(2):e00248-16. doi: 10.1128/mSphere.00248-16 (PMC5343172; doi:10.1128/mSphere.00248-16)

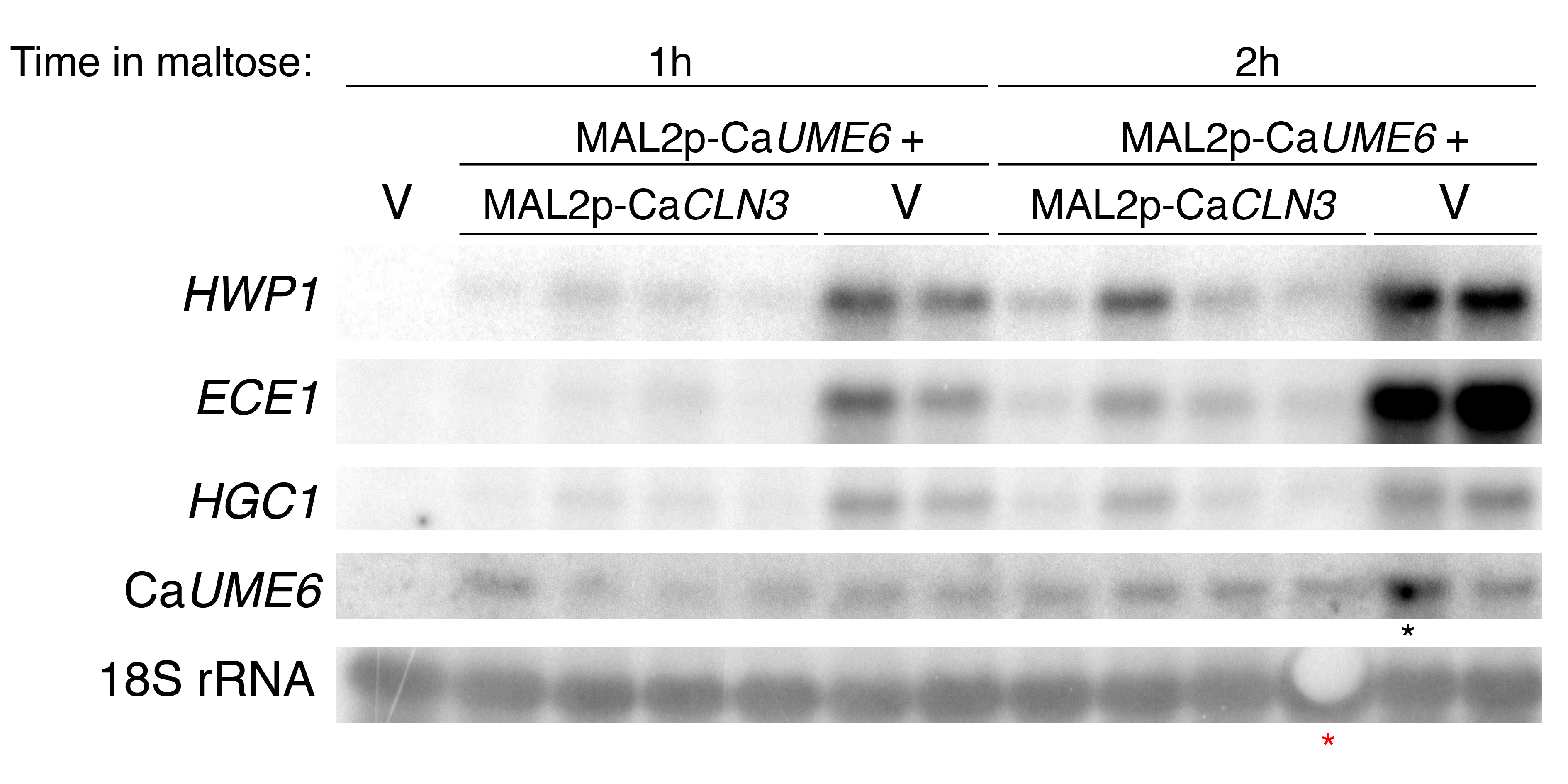

Supplement: FIG S1 [file sph002172249sf1.jpg]

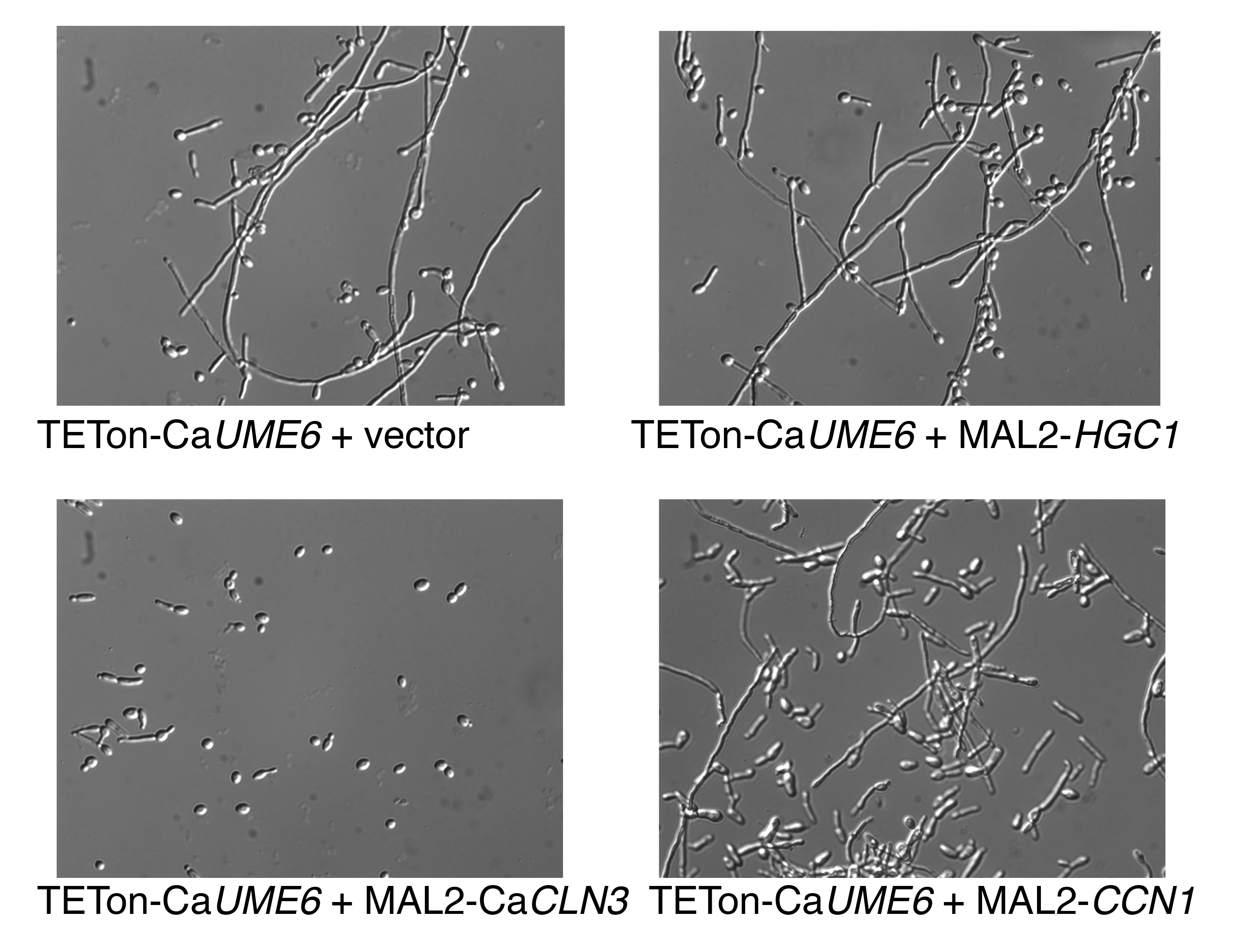

Supplement: FIG S2 [file sph002172249sf2.jpg]

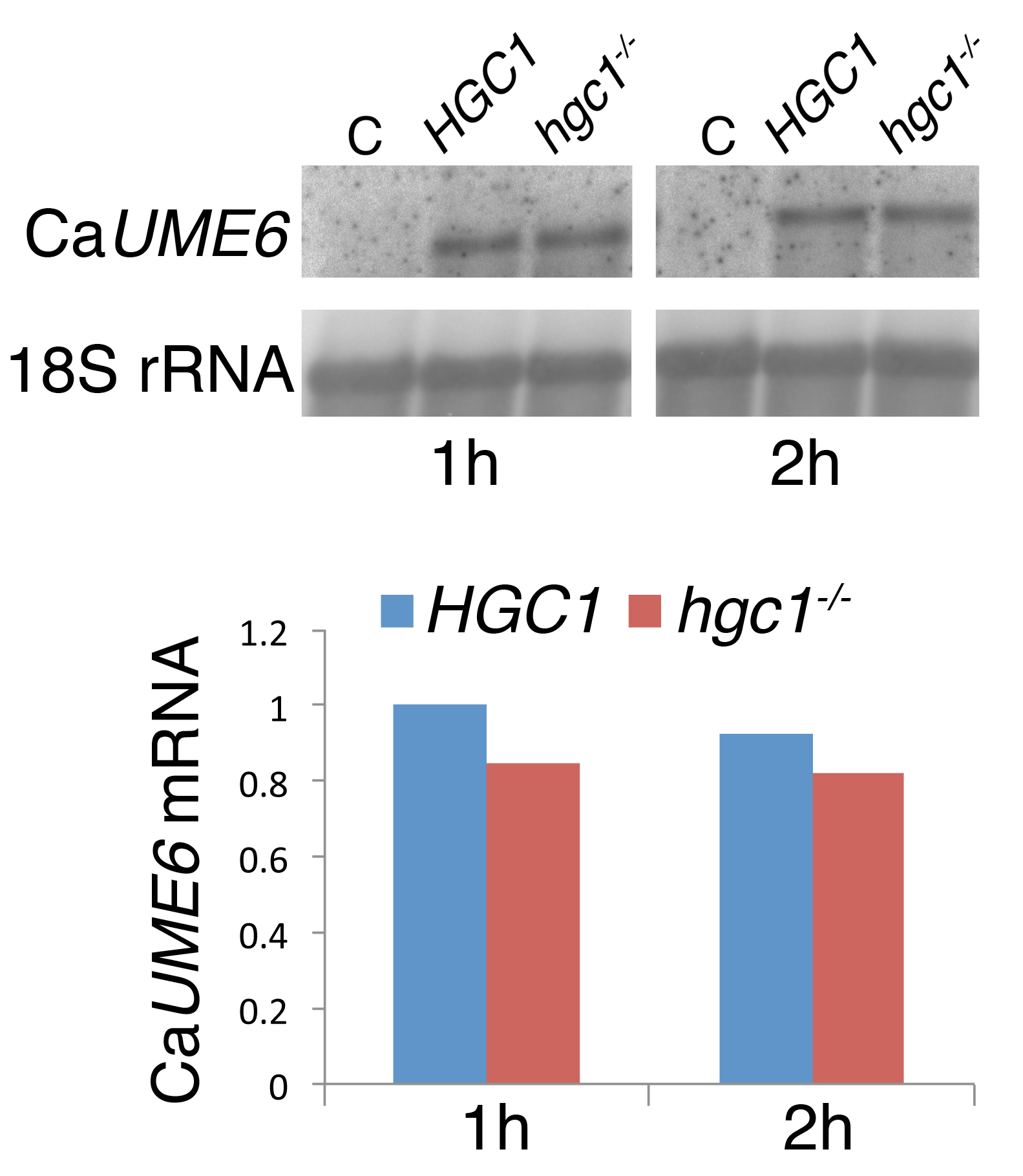

Supplement: FIG S3 [file sph002172249sf3.jpg]

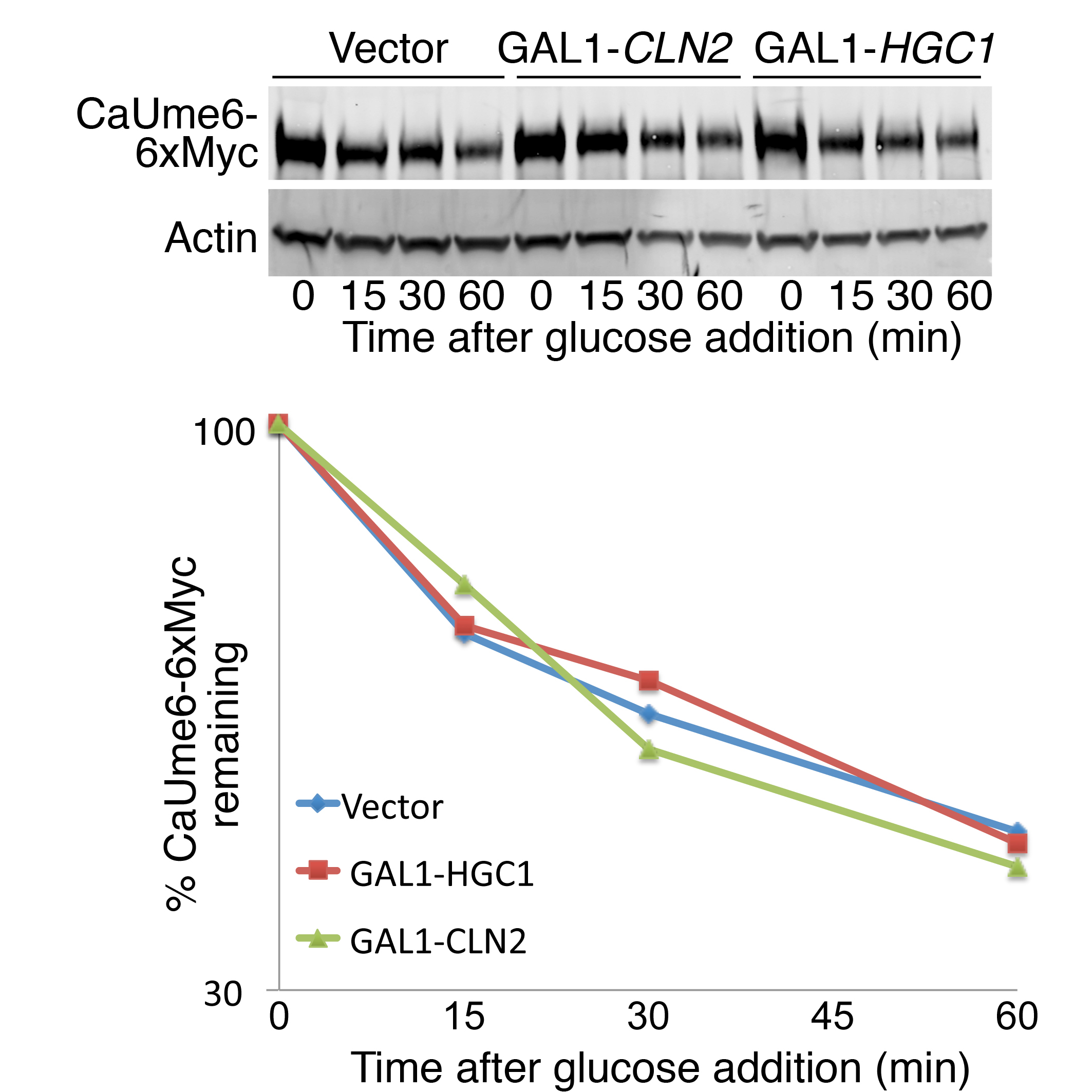

Supplement: FIG S4 [file sph002172249sf4.jpg]
